# Ultrasound‐Assisted Extraction of Mucilage: An Approach for the Full Utilization of Ora‐Pro‐Nóbis Leaves

**DOI:** 10.1111/1750-3841.71373

**Published:** 2026-08-17

**Authors:** Larissa Millena Girotto, Dayane Lilian Gallani Silva, Anderson Reginaldo Sampaio, Barbara Daniele Almeida Porciuncula, Beatriz Cervejeira Bolanho Barros

**Affiliations:** ^1^ Department of Technology State University of Maringá Umuarama Paraná Brazil; ^2^ Department of Physics State University of Maringá Maringá Paraná Brazil

**Keywords:** amino acids, *Pereskia aculeata* Miller, pseudoplastic behavior, rutin

## Abstract

Ora‐pro‐nóbis (OPN) leaves are remarkably high in mucilage content. Sustainable processing methods, such as ultrasound‐assisted extraction (UAE), can enhance the yield and functionality of OPN mucilage (OPNM). The resulting residue (OPNR) can be utilized to support circular economy approaches. This study aimed to determine the optimal conditions for OPNM extraction and subsequently characterize OPNR. OPNM was extracted by UAE at different power levels (200, 400, 600, and 800 W) and by conventional methods (water bath extraction at 25°C and 80°C). After extraction, OPNR was separated by filtration. OPNM samples showed lower protein contents (10.68–16.01 g 100 g^−1^) than OPNR samples (15.15–20.22 g 100 g^−1^) but higher water solubility (79.45–89.64 g 100 g^−1^), water absorption capacity (552.93–797.07 g 100 g^−1^), and total phenolic content (64.1–122.1 mg 100 g^−1^), resulting in higher antioxidant activity. Lightness values ranged from 35.97 to 41.72 (OPNM) and 38.55 to 44.79 (OPNR). OPNM samples had irregular and rough surfaces, high emulsifying capacity (600.68–621.31 mL oil g^−1^), and adequate emulsion stability (80.12%–86.90%). OPNM samples exhibited pseudoplastic behavior; the best balance between viscosity and structural integrity was achieved with the 400 W UAE treatment. OPNM also contained bioactive compounds, such as rutin and malic acid, as well as a complete profile of essential and nonessential amino acids, particularly histidine, glutamine, and aspartic acid. These results demonstrate the feasibility of the full utilization of OPN leaves, with UAE recommended as the most effective method for obtaining mucilage with enhanced functionality.

## 1 Introduction

Ora‐pro‐nóbis (OPN) (*Pereskia aculeata* Miller) is a nonconventional edible plant whose leaves are rich in proteins (25 g 100 g^−1^ dry basis) (Lima Jr. et al. 2013), dietary fibers (31.47 g 100 g^−1^ dry basis), and phenolic compounds (Silva et al. 2025). The leaves also contain mucilage (5 g 100 g^−1^ dry basis), mainly composed of polysaccharides, such as arabinogalactan, associated with protein fractions (Zeng and Lai 2016; Silveira et al. 2020). OPN mucilage (OPNM) exhibits emulsifying, thickening, and gelling properties, showing potential application in numerous food products (Lise et al. 2021; Oliveira et al. 2019).

Mucilage is usually extracted by simple methods involving wet grinding, filtration, precipitation, and centrifugation, with variations in extraction time and water temperature during grinding (Kobayasi et al. 2023; Masugossa et al. 2023). Due to the long extraction time (6–8 h) and the low efficiency of these methods, there is a need for alternative techniques, such as ultrasound‐assisted extraction (UAE). Ultrasonic waves induce cavitation, a process by which bubbles form and collapse near the cell walls of the material, promoting structural disruption and increasing the extraction rate (Flores‐Jiménez et al. 2019). Higher power levels may improve the mass transfer rate; however, intense liquid turbulence and circulation currents may degrade bioactive compounds, particularly phenolics (Liao et al. 2022). Thus, UAE may be a promising strategy to enhance mucilage recovery, but power levels should be carefully evaluated to minimize the loss of functional properties.

OPNM extraction by UAE has not yet been explored in the literature, nor has the extraction residue (OPNR) been evaluated. In light of circular economy principles, which promote efficient resource use and by‐product valorization, it is essential to characterize OPNR and investigate its utilization potential (Abdalla and Freire Sampaio 2018). Understanding the properties of this residue could enable its commercialization and future application in the formulation of food products. This study aimed to (i) extract OPNM using UAE at different power levels and compare the results with those of conventional extraction methods; (ii) assess the physicochemical characteristics, technological properties, and antioxidant activities of the resulting OPNM samples; and (iii) characterize the technological and antioxidant properties of OPNR to determine its application potential.

## 2 Materials and Methods

### 2.1 Materials

OPN leaves were manually harvested from the Medicinal Plant Farm at the Umuarama Regional Campus of the State University of Maringá (23°45′59″ S 53°19′30″ W), Paraná State, Brazil. The leaves were visually selected on the basis of their integrity and dark green coloration. Subsequently, the material was washed under running water and stored under refrigeration at 5°C until use.

The chemical reagents used in the analysis were ethanol (Anidrol), Folin–Ciocalteau reagent (Exodo Científica), gallic acid (Sigma‐Aldrich), Trolox (6‐hydroxy‐2,5,7,8‐tetramethylchroman‐2‐carboxylic acid, Merck), ABTS [2,2′‐azino‐bis(3‐ethylbenzothiazoline‐6‐sulfonic acid)] (Merck), DPPH (2,2‐diphenyl‐1‐picrylhydrazyl, Sigma‐Aldrich), 2,4,6‐tris(2‐pyridyl)‐*s*‐triazine (TPTZ, Sigma‐Aldrich), hydrochloric acid (Anidrol), phthalaldehyde (Sigma‐Aldrich), sodium borate (Lafan), Brij (Sigma‐Aldrich), mercaptoethanol (Neon), sodium acetate (Exodo Científica), acetic acid (Andirol), fomic acid (Merck), acetonitrile (Merck), and methanol (Merck).

Analytical standards used in ultra‐high‐performance liquid chromatography coupled with tandem mass spectrometry (UHPLC–MS/MS)—ferulic acid, *p*‐hydroxybenzoic acid, naringin, *p*‐coumaric acid, catechol, morin, isovanillin, quercetin, hydroxybenzaldehyde, naringenin, caffeine, resorcylic acid, nicotinic acid, syringaldehyde, chlorogenic acid, syringic acid, protocatechuic acid, vanillic acid, salicylic acid, vanillin, caffeic acid, coniferyl aldehyde, sinapic acid, syringaldazine, catechin, sinapaldehyde, luteolin, rutin, theobromine, epicatechin, baicalin, chrysin, quinic acid, malic acid, kaempferol, coumarin, and fumaric acid—were purchased from Sigma‐Aldrich (>99% purity). For free amino acid analysis, it was used as standards: aspartic acid, glutamic acid, asparagine, histidine, serine, alanine, glutamine, glycine, threonine, arginine, tyrosine, valine, methionine, tryptophan, phenylalanine, isoleucine, leucine, and lysine, from Sigma‐Aldrich (>99% purity).

### 2.2 Mucilage Extraction and Residue Obtention

Mucilage extraction was performed using conventional extraction and UAE, as shown in Figure S1.

Conventional extractions were carried out by grinding OPN leaves in water at room temperature (25°C) (CE25) and at 80°C (CE80). Both procedures were performed using a 1:3 sample‐to‐water ratio for 5 min, followed by incubation in a water bath (SL‐152, SOLAB Científica, Piracicaba, SP, Brazil) at 65°C for 6 h (Oliveira et al. 2019).

Probe‐type UAE (UCD‐950, BIOBASE, Jinan, Shandong, China) was performed according to Silva et al. (2022), with some modifications. First, OPN leaves were ground in a blender with water at room temperature (25°C) using a 1:3 sample‐to‐water ratio for 5 min. Ultrasound treatments were then applied for 30 min at 200 W (UAE200), 400 W (UAE400), 600 W (UAE600), and 800 W (UAE800). These power levels were selected according to the operating range of the equipment. Temperature was monitored throughout the extraction process, ranging from 37°C to 40°C.

For all methods, after the water bath or UAE steps, the material was subjected to vacuum filtration using a Büchner funnel lined with double‐layer organza fabric (40 mesh). The filtrate was centrifuged (SL‐706, SOLAB Científica, Piracicaba, Brazil) at 3000 rpm for 10 min, and the resulting supernatant was collected as mucilage (OPNM) (Oliveira et al. 2019). OPNM was subsequently freeze‐dried (LS3000, Terroni Equipamentos Científicos Ltda., São Carlos, Brazil), according to Lise et al. (2021).

The solid fraction retained during filtration, designated as OPNR, was dried in a forced‐air oven (MA 035/3BX, Marconi, Piracicaba, Brazil) at 100°C for 5 h, as described by Silva et al. (2024). Oven drying was selected due to its economic feasibility, which supports the potential large‐scale utilization of this by‐product (An et al. 2024). Dried samples were stored in sealed containers under refrigeration (5°C) until physicochemical, technological, and antioxidant analyses were performed.

### 2.3 Yield

The yields (*Y*) of OPNM and OPNR were calculated as the ratio of the weight of OPNM or OPNR (powder form, *W*_1_) and the weight of fresh OPN leaves (*W*_2_), according to

(1) $$Y=\frac{W_{1}}{W_{2}}\times 100$$

### 2.4 Physicochemical Characterization

Protein content, technological properties, colorimetry, total phenolic compounds, and antioxidant activity were evaluated on OPNM and OPNR samples obtained using different extraction methods. OPNM samples were also characterized by UHPLC–MS/MS, Fourier‐transform infrared spectroscopy (FTIR), and scanning electron microscopy and evaluated for emulsifying capacity, emulsion stability, and rheological properties. Additionally, the OPNM sample obtained under the best extraction conditions was analyzed for amino acid profile.

#### 2.4.1 Protein Content

Protein content was determined in triplicate for each treatment (OPNM and OPNR) using the micro‐Kjeldahl method and a nitrogen‐to‐protein conversion factor of 6.25 (AOAC 2005).

#### 2.4.2 Technological Properties

The water solubility index (WSI), water absorption index (WAI), and oil absorption index (OAI) were determined according to the methods described by Silva et al. (2024). Results are expressed as g of soluble solids per 100 g of sample.

#### 2.4.3 Color Parameters

Colorimetric analysis was performed using a colorimeter (Konica Minolta CR‐10, Tokyo, Japan). Lightness (L*), redness–greenness (a*), and yellowness–blueness (b*) were measured (Konica Minolta 2022).

#### 2.4.4 Total Phenolic Content (TPC) and Antioxidant Activity

Samples were diluted at a ratio of 1:40 (w v^−1^) in a 50% ethanolic solution (v v^−1^) and agitated in an orbital shaker (Tecnal TE‐141, Piracicaba, Brazil) at 200 rpm for 1 h. Subsequently, the samples were centrifuged at 3000 rpm for 10 min (Lise et al. 2021).

The extracts were evaluated for TPC using the Folin–Ciocalteu method (Shahidi and Zhong 2015). Calibration curves (*R* ^2^ > 0.99) were prepared using gallic acid solutions ranging from 25 to 500 mg L^−1^, and the results are expressed as gallic acid equivalents (GAE).

Antioxidant capacity was determined using the free radical scavenging assays ABTS and DPPH and the ferric reducing antioxidant power (FRAP) assay, according to Thaipong et al. (2006), Ravichandran et al. (2012), and Benzie and Strain (1996), respectively. Calibration curves (*R* ^2^ ≥ 0.99) were constructed using Trolox at concentrations ranging from 0.10 to 0.80 mM, and the results are expressed as Trolox equivalents.

#### 2.4.5 UHPLC–MS/MS Analysis

OPNM samples were analyzed by UHPLC–MS/MS (Shimadzu 8050 MS and Nexera X2 HPLC, Tokyo, Japan) using a C18 column (5 µm, 150 × 4.6 mm, Shimadzu). The mobile phases were operated in linear gradient mode at a flow rate of 0.5 mL min^−1^, using Milli‐Q water supplemented with (i) 0.1% (v v^−1^) formic acid and (ii) MS‐grade methanol, according to the following gradient program: 1–9 min, 20% B; 10–15 min, 40% B; and 16–30 min, 10% B. The autosampler and column temperatures were maintained at 40°C, and the injection volume was 1 µL. The MS/MS detector was operated in scanning mode for 15 min using an electrospray ionization source in both positive and negative ionization modes with multiple reaction monitoring. The nebulizing gas flow rate (3 L min^−1^), drying gas flow rate (10 L min^−1^), interface temperature (300°C), and desolvation line temperature (526°C) were maintained throughout all analyses. The standard compounds (described in Section 2.1) were detected and quantified using LabSolutions Insight software (Shimadzu) (Bertoco Jr. et al. 2025).

#### 2.4.6 Emulsifying Capacity and Emulsion Stability

The emulsifying capacity and emulsion stability of the OPNM samples were evaluated according to the method described by Lima Jr. et al. (2013). The results are expressed as mL of emulsified oil per gram of OPNM.

#### 2.4.7 Rheological Properties

Rheological analyses were performed using a Thermo Scientific rheometer (HAAKE MARS II, Waltham, MA, USA). The cone‐and‐plate geometry (CP60/1Ti shear sensor; 60 mm diameter and 1° cone angle) was used. Shear stress (*τ*) data as a function of shear rate (*γ̇*) were recorded in controlled‐rate rotational mode, with shear rates ranging from 5.0 to 300 s^−1^ at a constant temperature of 25°C (Rigoto et al. 2018).

Apparent viscosity (*η*_ap_) data were fitted as a function of shear rate using the Ostwald–de Waele model (power law), as shown in Equation (2), where *K* is the consistency index and *n* is the flow behavior index:

(2) $$\eta_{\mathrm{ap}}=K\times {\dot{\gamma }}^{n-1}$$

#### 2.4.8 FTIR

OPNM samples were analyzed by FTIR using an Agilent Cary 630 spectrometer (Agilent Technologies, Santa Clara, CA, USA) equipped with an attenuated total reflectance module. Spectra were collected using 45 scans at a resolution of 4 cm^−1^ over the spectral range of 4000–400 cm^−1^.

#### 2.4.9 Scanning Electron Microscopy

OPNM samples were mounted on aluminum stubs, sputter‐coated with gold, and analyzed using scanning electron microscopy (Quanta 250, FEI Company, Hillsboro, OR, USA), with the accelerating voltage set at 12.5 kV. Images were acquired at magnifications of 500× and 2000×.

#### 2.4.10 Amino Acid Profile

The OPNM sample that showed the best performance in the characterization analyses (UAE400) was selected for amino acid evaluation, as it represents the most promising sample for further applications. The sample was subjected to acid hydrolysis using 6 mol L^−1^ hydrochloric acid at a sample‐to‐acid ratio of 1:25 (w v^−1^). The mixture was autoclaved at 121°C for 8 min. The hydrolyzate was then filtered through cellulose filter paper with a pore size of 0.45 µm, and the reaction medium was evaporated in a sand bath at 110°C. The resulting samples were washed five times with 5 mL of Milli‐Q water, with the washings being evaporated under the same conditions. The obtained residue was resuspended in 10 mL of Milli‐Q water and homogenized in an ultrasonic bath for 3 min. Finally, the solution was filtered again through cellulose filter paper (0.45 µm) and transferred to 25 mL volumetric flasks, and the pH of the extract was adjusted to 4.5 (Denardi‐Souza et al. 2018).

The extract was analyzed by HPLC (LC‐20A, Shimadzu Corporation, Kyoto, Japan) equipped with a fluorescence detector (RF‐20A, Shimadzu), following a method adapted from Corleto et al. (2019). The amino acids were derivatized using a mixture of phthalaldehyde, sodium borate, Brij, and mercaptoethanol. The elution gradient consisted of 20 mM sodium acetate (pH 5.40) (Mobile Phase A) and a mixture of 45% acetonitrile, 45% methanol, and 10% water (Mobile Phase B), applied as follows: 1–50 min, 20% B; 50–80 min, 40% B; 80–120 min, 40% B; 120–125 min, 90% B; 125–137 min, 90% B; and 137–140 min, 20% B.

A shim‐pack GIST C8 column (4.6 × 250 mm, Shimadzu) maintained at 30°C was used for amino acid separation, with an injection volume of 5 µL and a flow rate of 0.5 mL min^−1^. The fluorescence detector (RF‐20A) was operated at excitation and emission wavelengths of 340 and 455 nm, respectively. The amino acid standards (Section 2.1) were used to obtain calibration curves (0.1–5.0 µg mL^−1^, *R* ^2^ > 0.99). The results were expressed as mg g^−1^ of sample.

### 2.5 Data Analysis

All extractions were performed in triplicate, and all analytical determinations were conducted in triplicate. Results are expressed as mean ± standard deviation. Data were subjected to one‐way analysis of variance (ANOVA), and differences among means were evaluated using Tukey's test at *p* < 0.05. Statistical analyses were performed using Statistica software version 7.0 (StatSoft Inc., Tulsa, OK, USA).

## 3 Results and Discussion

### 3.1 Yield

Table 1 presents the yields of OPNM and OPNR samples obtained by different methods.

**TABLE 1:** Yield, protein content, and technological properties of ora‐pro‐nóbis mucilage and the corresponding extraction residues obtained using different methods.

Sample	Yield (% w w^−1^)	Protein content (g 100 g^−1^)	Water solubility index (g 100 g^−1^)	Water absorption index (g 100 g^−1^)	Oil absorption index (g 100 g^−1^)
**Mucilage**					
CE25	2.988 ± 0.215^a^	14.630 ± 0.147^a^	89.42 ± 0.554^a^	610.020 ± 0.497^c^	351.281 ± 0.289^d^
CE80	3.725 ± 0.250^a^	15.706 ± 0.188^a^	87.551 ± 0.730^a^	619.980 ± 0.593^c^	359.523 ± 0.384^c^
UAE200	3.201 ± 0.554^a^	16.012 ± 1.034^a^	82.755 ± 0.276^bc^	719.842 ± 0.953^b^	366.131 ± 0.256^b^
UAE400	3.314 ± 0.279^a^	14.540 ± 0.342^a^	87.225 ± 0.906^ab^	797.072 ± 0.902^a^	361.311 ± 0.546^bc^
UAE600	3.189 ± 0.257^a^	10.680 ± 0.131^b^	79.451 ± 0.558^c^	601.870 ± 0.704^c^	369.730 ± 0.205^b^
UAE800	2.713 ± 0.287^a^	10.973 ± 0.699^b^	82.421 ± 2.501^c^	552.931 ± 0.971^d^	477.010 ± 0.216^a^
**Residue**					
CE25	9.891 ± 0.006^c^	15.152 ± 0.444^b^	5.964 ± 0.556^a^	19.385 ± 1.641^bc^	372.912 ± 0.989^b^
CE80	13.382 ± 0.070^a^	16.051 ± 0.174^b^	5.339 ± 0.311^a^	24.363 ± 0.028^a^	306.524 ± 1.032^c^
UAE200	12.418 ± 0.070^b^	15.738 ± 1.538^b^	4.915 ± 0.713^a^	21.776 ± 0.035^ab^	402.331 ± 1.421^a^
UAE400	12.382 ± 0.070^b^	18.669 ± 0.142^a^	5.463 ± 0.198^a^	22.606 ± 0.485^ab^	393.623 ± 1.252^ab^
UAE600	12.423 ± 0.071^b^	15.896 ± 0.591^b^	4.641 ± 0.181^a^	15.839 ± 0.968^c^	407.405 ± 1.513^a^
UAE800	6.214 ± 0.071^d^	20.224 ± 0.827^a^	1.985 ± 0.241^b^	22.565 ± 0.826^ab^	415.314 ± 1.670^a^

*Note*: Results are the mean ± standard deviation of triplicate assays. Means within columns followed by the same letter are not significantly different by Tukey's test (*p* ≤ 0.05).

Abbreviations: CE25, conventional extraction at 25°C; CE80, conventional extraction at 80°C; UAE200, ultrasound‐assisted extraction at 200 W; UAE400, ultrasound‐assisted extraction at 400 W; UAE600, ultrasound‐assisted extraction at 600 W; UAE800, ultrasound‐assisted extraction at 800 W.

OPNM extraction yields ranged from 2.7% to 3.7% (w w^−1^), with no significant differences among treatments. These values are close to those reported by Kobayasi et al. (2023), who obtained 4.18% (w w^−1^) yield, and higher than those described by Martin et al. (2017), who reported a yield of 1.6% (w w^−1^).

OPNR yields ranged from 6.2% to 13.4% (w w^−1^). The highest yield was obtained in the CE80 treatment, followed by UAE treatments at power levels between 200 and 600 W. The result for CE80 was similar to that reported by Sommer et al. (2022), who obtained 13% (w w) yield in the production of OPN meal. The lowest residue yield (6.21%) observed in the UAE800 treatment may be attributed to the intense cavitation generated at the high ultrasonic power of 800 W. Such conditions can induce tissue disintegration and particle size reduction (Liu et al. 2022), potentially increasing the retention of fine particles on the organza fabric used in the filtration step.

### 3.2 Protein Content

The protein content of OPNM (Table 1) ranged from 10.68 to 16.01 g 100 g^−1^. The highest values were found in CE25, CE80, UAE200, and UAE400 samples, without significant differences among them. Thus, the application of UAE at low powers (200 and 400 W) proved to be favorable for the extraction of OPN proteins, requiring less time (30 min) than conventional methods (360 min). The remarkable effectiveness of UAE in extracting food proteins can be attributed to the rupture of the cell wall caused by the effect of shear forces, micro‐jets, and shock waves, as well as to the formation of hydroxyl radicals promoted by the collapse of cavitation bubbles, which promotes the extraction of protein chains (Sengar et al. 2022). The protein contents found in the current study were similar to those reported for OPNM (8.89 g 100 g^−1^) by Oliveira et al. (2019) and for an OPN extract (13.39 g 100 g^−1^) by Maciel et al. (2021).

For OPNR, protein content ranged from 15.15 to 20.22 g 100 g^−1^, with the highest values observed in UAE400 and UAE800 samples, which did not differ significantly from each other. Arena et al. ((2023) prepared OPN protein concentrates containing 24.12 to 31.63 g 100 g^−1^ of protein.

The high protein content found in both OPNM and OPNR is noteworthy, as proteins contribute not only to nutritional enrichment but also to functional performance. In combination with polysaccharides such as arabinogalactans, proteins can enhance various technological properties of processed foods, including emulsifying and foaming capacity and oil and water retention (Vieira et al. 2021).

### 3.3 Technological Properties

Regarding technological properties (Table 1), OPNM solubility ranged from 79.45 to 89.64 g 100 g^−1^. The highest values were observed for the CE25, CE80, and UAE400 samples, which did not differ significantly from one another. These results were higher than those reported by Lise (2021), who found a solubility of 20.68 g 100 g^−1^ for OPNM. This discrepancy may be attributed to variations in the composition of plant matrices and differences between extraction (Antigo et al. 2020).

The WAI values of OPNM ranged from 552.93 to 797.07 g 100 g^−1^, with the highest value observed for UAE400. This value was higher than those reported by Gemede et al. (2018) for indigenous Ethiopian okra mucilage (270 g 100 g^−1^) and by Monrroy et al. (2017) for *Opuntia cochenillifera* (L.) Miller mucilage (278 g 100 g^−1^). These findings support previous reports that plants belonging to the family Cactaceae, such as OPN, produce mucilage with a high capacity for water absorption (Petera et al. 2015).

Comparison of the results between extraction conditions revealed a reduction in WSI and WAI values at higher UAE power levels (600 and 800 W). This decrease may be attributed to the hydrolysis of arabinogalactan polysaccharides into oligosaccharides, reducing their ability to interact with mucilage proteins and water (Wang et al. 2022). Moreover, cavitation at intermediate power (400 W) might have enhanced interactions between protein and water molecules by disrupting hydrogen bonds and hydrophobic interactions, whereas higher power levels might have promoted the exposure of internal hydrophobic groups, thereby decreasing protein solubility (Sengar et al. 2022).

WSI and WAI values were lower in OPNR than in OPNM. The solubility of OPNR ranged from 1.98 to 5.96 g 100 g^−1^, with the UAE800 sample showing the lowest solubility and being the only one that differed significantly from the others. The WAI values of OPNR samples ranged from 15.84 to 24.36 g 100 g^−1^, with the highest values observed for CE80, UAE200, UAE400, and UAE800, which did not differ from one another. The low values of these parameters compared to OPNM may be related to the higher content of insoluble fibers in OPNR. OPN leaves are known to contain up to six times more insoluble fiber than soluble fiber (Silva et al. 2025).

The OAI of OPNM ranged from 351.28 to 477.01 g 100 g^−1^, whereas, in OPNR, values ranged from 306.5 to 415.3 g 100 g^−1^. The UAE800 sample showed the highest OAI among mucilages, which may be associated with increased cavitation and the consequent exposure of hydrophobic groups (Janer et al. 2020). Among OPNR samples, those exposed to ultrasonication exhibited the highest OAI values. All OAI results, for both OPNM and OPNR, were higher than those reported by Monrroy et al. (2017) for mucilage from *O. cochenillifera* (180 g 100 g^−1^).

Overall, OPNM demonstrates strong application potential due to its bifunctional character, exhibiting both hydrophilic and hydrophobic properties that favor its use in diverse products and formulations. For instance, OPNM can be used as a humectant in bakery products, such as bread, cakes, and pastries, improving thickening and increasing viscosity (Aracava et al. 2022).

Although OPNR showed lower water absorption capacity than OPNM, its application remains viable, particularly because its OAI values were comparable to those of OPNM. This characteristic may be especially advantageous in meat products, contributing to improved juiciness and palatability (Nishimura 2021). OPNR can also be used in salad dressings, mayonnaise, and plant‐based products as a protein source (Aracava et al. 2022).

### 3.4 Color Parameters

The color parameters of OPNM and OPNR are presented in Table 2.

**TABLE 2:** Colorimetric analysis of ora‐pro‐nóbis mucilage and the corresponding extraction residues obtained using different methods.

Sample	Color parameter		
	L*	a*	b*
Mucilage			
CE25	40.207 ± 0.151^b^	0.776 ± 0.065^c^	7.510 ± 1.187^ab^
CE80	35.973 ± 0.040^d^	0.337 ± 0.012^d^	4.313 ± 0.059^c^
UAE200	37.427 ± 0.157^c^	−0.600 ± 0.010^e^	6.480 ± 0.036^ab^
UAE400	39.760 ± 0.689^b^	1.513 ± 0.035^b^	7.357 ± 1.328^ab^
UAE600	41.740 ± 0.072^a^	−1.033 ± 0.021^f^	8.640 ± 0.217^a^
UAE800	41.723 ± 0.159^a^	3.33 ± 0.051^a^	8.030 ± 0.122^ab^
**Residue**			
CE25	43.270 ± 0.733^c^	0.370 ± 0.056^b^	5.873 ± 0.297^b^
CE80	39.493 ± 0.316^de^	0.140 ± 0.050^c^	5.496 ± 0.233^b^
UAE200	46.410 ± 0.305^a^	0.730 ± 0.072^a^	7.597 ± 0.238^a^
UAE400	40.013 ± 0.341^d^	0.657 ± 0.065^a^	7.457 ± 0.337^a^
UAE600	38.550 ± 0.514^e^	0.687 ± 0.049^a^	5.313 ± 0.125^b^
UAE800	44.797 ± 0.099^b^	0.706 ± 0.015^a^	7.023 ± 0.838^a^

*Note*: Results are the mean ± standard deviation of triplicate assays. Means within columns followed by the same letter are not significantly different by Tukey's test (*p* ≤ 0.05).

Abbreviations: CE25, conventional extraction at 25°C; CE80, conventional extraction at 80°C; UAE200, ultrasound‐assisted extraction at 200 W; UAE400, ultrasound‐assisted extraction at 400 W; UAE600, ultrasound‐assisted extraction at 600 W; UAE800, ultrasound‐assisted extraction at 800 W.

OPNM and OPNR showed lightness (L*) values ranging from 35.97 to 41.72 and 38.55 to 44.79, respectively. These results indicate a tendency toward darker coloration, as previously reported by Sobrinho et al. (2015), who found an L* value of 34.37 for OPN powder.

Among OPNMs, the lowest L* value was observed in the CE80 sample (35.97). Among OPNRs, the lowest L* values were found in CE80 and UAE600 (39.49 and 38.55, respectively). Heat treatment may explain the reduction in lightness, as the CE80 method involved heating at 80°C for 6 h. Thermal processing can denature proteins that protect chlorophyll, leading to loss of magnesium ions and the formation of pheophytin, which results in an olive‐green color and reduced lightness (Long et al. 2025).

The greenness–redness (a*) values of OPNM ranged from −1.03 to 3.93, with the lowest values observed for UAE200 and UAE600. The remaining mucilage samples (CE25, CE80, UAE400, and UAE800), as well as all OPNR samples, showed a* values between 0.14 and 3.93, indicating a tendency toward green coloration.

Blueness–yellowness (b*) values ranged from 5.31 to 7.59 for OPNM and from 4.31 to 8.64 for OPNR, indicating a tendency toward yellow coloration. Similarly, Arena et al. (2023) reported b* values between 7.91 and 8.68 for OPN powder. In the current study, a yellow color was observed in all samples, being particularly pronounced in those subjected to ultrasonication, possibly due to the facilitated release of carotenoids by the disruption of internal leaf structures (Siqueira et al. 2024).

### 3.5 TPC and Antioxidant Activity

Table 3 describes the TPC and antioxidant activity of OPNM and OPNR.

**TABLE 3:** Total phenolic compound content (TPC) and antioxidant activity of ora‐pro‐nóbis mucilage and the corresponding extraction residues obtained using different methods.

Sample	TPC (mg GAE g^−1^)	Antioxidant capacity (µM Trolox equivalents 100 g^−1^)		
		FRAP	ABTS	DPPH
Mucilage				
CE25	55.306 ± 0.192^d^	52.661 ± 1.204^c^	35.416 ± 0.805^d^	23.367 ± 0.614^d^
CE80	43.955 ± 1.090^e^	45.345 ± 0.595^d^	33.084 ± 0.821^d^	23.940 ± 0.360^d^
UAE200	64.098 ± 0.898^c^	26.845 ± 0.275^e^	28.505 ± 0.799^e^	19.722 ± 0.129^e^
UAE400	105.694 ± 2.248^b^	71.196 ± 0.296^b^	52.521 ± 0.712^c^	29.912 ± 0.085^c^
UAE600	106.096 ± 1.123^b^	73.779 ± 1.110^bc^	68.138 ± 1.219^b^	32.111 ± 0.937^b^
UAE800	122.104 ± 0.650^a^	84.653 ± 1.047^a^	77.726 ± 0.607^a^	41.549 ± 0.423^a^
**Residue**				
CE25	19.793 ± 1.282^a^	23.108 ± 0.385^a^	2.767 ± 0.071^a^	1.816 ± 0.035^a^
CE80	17.946 ± 0.587^ab^	23.449 ± 0.698^a^	2.502 ± 0.068^b^	1.846 ± 0.054^a^
UAE200	15.577 ± 0.540^c^	22.464 ± 0.816^a^	1.888 ± 0.103^c^	1.827 ± 0.066^a^
UAE400	17.037 ± 0.807^bc^	19.475 ± 0.945^b^	1.897 ± 0.070^c^	1.579 ± 0.048^c^
UAE600	16.692 ± 0.573^bc^	18.117 ± 0.923^bc^	1.939 ± 0.149^c^	1372 ± 0.022^b^
UAE800	12.523 ± 0.473^d^	17.242 ± 0.870^bc^	1.479 ± 0.032^d^	1.289 ± 0.116^b^

*Note*: Results are the mean ± standard deviation of triplicate assays. Means within columns followed by the same letter are not significantly different by Tukey's test (*p* ≤ 0.05).

Abbreviations: CE25, conventional extraction at 25°C; CE80, conventional extraction at 80°C; GAE, gallic acid equivalents; UAE200, ultrasound‐assisted extraction at 200 W; UAE400, ultrasound‐assisted extraction at 400 W; UAE600, ultrasound‐assisted extraction at 600 W; UAE800, ultrasound‐assisted extraction at 800 W.

Among OPNM samples, the highest TPCs were obtained when using UAE. Total phenolics increased with increasing power, ranging from 43.95 to 122.10 mg GAE g^−1^. The highest TPC was observed for UAE800. The recorded values were higher than those reported by Messina et al. (2021) for mucilage of *Opuntia ficus‐indica* Miller (12.57 mg GAE g^−1^).

UAE is an efficient and environmentally friendly option for the extraction of compounds, promoting reproducibility, speed, and low costs (Chemat et al. 2017). According to Bernardo et al. (2016), UAE increases the purity of hydrocolloids, as it does not damage the matrix or hinder the passage of other compounds, in addition to favoring the extraction of compounds with antioxidant action, compared with conventional extraction methods (Bertoco Jr. et al. 2025). Cavitation bubbles can rupture and alter the structure of high molecular weight compounds such as lignin, contributing to the release of phenolics (Hedayati et al. 2021).

The increase in the extraction of phenolic compounds in OPNM samples led to an increase in antioxidant activity, as measured by the ABTS, DPPH, and FRAP methods. Overall, samples extracted by UAE showed higher antioxidant activity. UAE also increased (15%) the antioxidant activity of fenugreek seed mucilage compared with conventional methods (Niknam et al. 2020). In the current study, the increase in ABTS activity was even greater, reaching approximately 60% when UAE800 was compared with CE25. The high phenolics content and antioxidant activity of the UAE800 sample can be explained by the fact that higher ultrasound power increases turbulence during extraction, favoring solvent permeation and compound extraction. Low ultrasound power, on the other hand, may not provide sufficient energy for the extraction of these compounds (Niknam et al. 2020). The low phenolics content and antioxidant activity of samples extracted using a water bath may be attributed to the prolonged exposure to high temperatures, which can degrade antioxidant compounds (Schafranski et al. 2019).

Among residues obtained after mucilage extraction, the highest TPC was observed in CE25 and CE80 samples, which did not differ significantly from each other. The lowest content was observed in UAE800. The OPNR sample obtained by CE25 had the highest antioxidant activity according to all evaluation methods, not differing from the sample obtained by CE80 or UAE200 in FRAP and DPPH assays. Ciríaco et al. (2023) reported a lower TPC (10.01 mg GAE g^−1^) but higher antioxidant activity (6.30 µM Trolox equivalents 100 g^−1^, ABTS) in oven‐dried OPN leaves.

Total phenolic levels were 2 to 10 times higher in OPNM samples than in OPNR samples, which was reflected in antioxidant activity. This result was expected, as OPNR is a by‐product of OPNM extraction. Thus, the higher the extraction of antioxidant compounds in mucilage, the lower their content in residues, and vice versa. Such a relationship is evident when comparing the results for CE25 and CE80, in which the resulting OPNMs had the lowest phenolic levels, and the respective OPNRs exhibited the highest levels.

### 3.6 OPNM Characterization

Given the good technological properties (WAI, OAI, and WSI) and high antioxidant potential of OPNM, the samples were further characterized, as described below.

#### 3.6.1 Composition by UHPLC–MS/MS

Among the 35 standard compounds used in UHPLC–MS/MS, 13 were detected in OPNM samples, including four phenolic acids (chlorogenic acid, protocatechuic acid, ferulic acid, and caffeic acid), three phenolic aldehydes (isovanillin, hydroxybenzaldehyde, and vanillin), three flavonoids (rutin, naringenin, and quercetin), two carboxylic acids (malic and fumaric), and one lactone (coumarin). For all samples, naringenin, quercetin, and coumarin were detected at concentrations below the quantification limit (<1 µg g^−1^), whereas rutin content was above the maximum quantification limit (>500 µg g^−1^). The quantification of the other phenolic compounds is shown in Table 4.

**TABLE 4:** Composition of ora‐pro‐nóbis mucilage obtained using different extraction methods, as analyzed by ultra‐high‐performance liquid chromatography coupled with tandem mass spectrometry (UHPLC–MS/MS).

Sample	Compounds (µg g^−1^)								
	Isovanillin	Hydroxybenzaldehyde	Chlorogenic acid	Protocatechuic acid	Vanillin	Ferulic acid	Caffeic acid	Malic acid	Fumaric acid
CE25	7.207 ± 0.236^bc^	3.677 ± 0.002^a^	1.738 ± 0.016^cd^	3.261 ± 0.129^a^	22.903 ± 0.751^d^	4.908 ± 0.523^b^	33.408 ± 0.432^b^	161.105 ± 1.301^c^	15.591 ± 0.485^ab^
CE80	7.695 ± 0.084^b^	2.846 ± 0.003^c^	2.727 ± 0.245^a^	3.068 ± 0.050^a^	21.821 ± 0.369^d^	5.450 ± 0.044^ab^	34.971 ± 1.089^b^	179.029 ± 2.339^c^	14.727 ± 0.262^abc^
UAE200	7.704 ± 0.223^b^	2.481 ± 0.137^d^	1.577 ± 0.179^cd^	1.892 ± 0.660^b^	28.084 ± 0.225^b^	4.428 ± 0.302^b^	15.700 ± 0.020^c^	168.448 ± 1.916^c^	10.048 ± 0.530^d^
UAE400	6.690 ± 0.023^c^	2.819 ± 0.073^c^	1.275 ± 0.017^d^	2.269 ± 0.225^b^	27.346 ± 0.028^bc^	4.341 ± 0.258^b^	16.594 ± 0.230^c^	178.999 ± 0.333^c^	12.662 ± 0.881^c^
UAE600	8.454 ± 0.142^a^	3.406 ± 0.060^b^	2.032 ± 0.024^bc^	3.121 ± 0.016^a^	25.831 ± 0.043^c^	4.341 ± 0.258^b^	44.229 ± 0.046^a^	231.036 ± 2.901b	13.333 ± 0.588^bc^
UAE800	12.293 ± 0.032^a^	3.846 ± 0.013^a^	2.324 ± 0.068^ab^	3.010 ± 0.135^a^	41.015 ± 0.610^a^	5.758 ± 0.030^a^	35.235 ± 0.828^b^	320.044 ± 14.382^a^	12.662 ± 0.881^c^

*Note*: Results are the mean ± standard deviation of triplicate assays. Means within columns followed by the same letter are not significantly different by Tukey's test (*p *≤ 0.05).

Abbreviations: CE25, conventional extraction at 25°C; CE80, conventional extraction at 80°C; UAE200, ultrasound‐assisted extraction at 200 W; UAE400, ultrasound‐assisted extraction at 400 W; UAE600, ultrasound‐assisted extraction at 600 W; UAE800, ultrasound‐assisted extraction at 800 W.

No previous study had identified or quantified the phenolic compounds present in OPNM; however, some studies reported the presence of quercetin (Rahman et al. 2017), caffeic acid (Cabañas‐García et al. 2019), ferulic acid (Jiménez‐Aspee et al. 2014), and protocatechuic acid (Cabañas‐García et al. 2019) in OPN leaves.

Rutin was the major compound (>500 µg g^−1^), followed by malic acid (161.105–320.044 µg g^−1^). Rutin is a flavonoid with strong antioxidant activity that protects against cellular oxidative damage, contributes to the reduction of adipose tissue, and exerts hepatoprotective effects (Ghanbari‐Movahed et al. 2022). Malic acid has several applications in the pharmaceutical and food industries, including as an antioxidant, acidulant, and flavor enhancer (Jiayuan et al. 2026).

The UAE800 sample was found to contain the highest contents of bioactive compounds, except for caffeic acid. However, the content of some compounds did not differ between extraction conditions, such as isovanillin (UAE600 and UAE800), hydroxybenzaldehyde (CE25 and UAE800), chlorogenic acid (CE80 and UAE800), protocatechuic acid (CE25, CE80, UAE600, and UAE800), and fumaric acid (CE25, CE80, and UAE800).

UAE800 resulted in a 60% increase in isovanillin, 75% increase in chlorogenic acid, and 90% increase in malic acid levels compared with conventional extraction methods (CE25 and CE80); these results corroborate those obtained for TPC and antioxidant activity (Table 3). Similarly, Goldsmith et al. (2017) reported a 66% increase in phenolic contents extracted from olive pomace with the use of UAE at high powers. This effect may be attributed to the greater solvent penetration at high sonication power, promoting compound recovery (Osorio‐Tobón 2020). The findings demonstrate the efficacy of UAE, especially at high powers, in releasing bioactive compounds from OPNM, underscoring the potential of this material as a natural source of antioxidants.

#### 3.6.2 Emulsifying Capacity and Emulsion Stability

The emulsifying capacity and emulsion stability of OPNM samples are reported in Table 5.

**TABLE 5:** Emulsifying capacity, emulsion stability, and rheological properties of ora‐pro‐nóbis mucilage obtained using different extraction methods.

Sample	Emulsifying properties		Rheological properties		
	Emulsifying capacity (mL oil g^−1^)	Emulsion stability (% w w^−1^)	$\eta_{\mathrm{ap}}$ (mPa s)	*K* (Pa s)*^n^*	*n*
CE25	610.048 ± 2.586^cd^	80.60 ± 0.070^c^	125.05 ± 1.95^b^	7.08 ± 0.08^a^	0.25 ± 0.01^d^
CE80	606.848 ± 1.894^d^	80.31 ± 0.212^c^	30.08 ± 0.98^f^	0.16 ± 0.01^e^	0.75 ± 0.01^a^
UAE200	600.686 ± 1.532^e^	80.12 ± 0.141^c^	99.83 ± 1.51^d^	2.27 ± 0.18^c^	0.49 ± 0.02^b^
UAE400 UAE600	621.315 ± 0.145^a^ 615.141 ± 0.129^bc^	86.90 ± 0.282^a^ 84.53 ± 0.141^b^	146.31 ± 0.78^a^ 112.02 ± 2.05^c^	4.89 ± 0.03^b^ 2.26 ± 0.05^c^	0.39 ± 0.01^c^ 0.48 ± 0.02^b^
UAE800	617.612 ± 0.132^ab^	86.31 ± 0.353^a^	44.84 ± 0.59^e^	0.57 ± 0.04^d^	0.78 ± 0.03^a^

*Note: η*_ap_, apparent viscosity; *K*, consistency index; *n*, melt flow index. Results are the mean ± standard deviation of triplicate assays. Means within columns followed by the same letter are not significantly different by Tukey's test (*p* ≤ 0.05).

Abbreviations: CE25, conventional extraction at 25°C; CE80, conventional extraction at 80°C; UAE200, ultrasound‐assisted extraction at 200 W; UAE400, ultrasound‐assisted extraction at 400 W; UAE600, ultrasound‐assisted extraction at 600 W; UAE800, ultrasound‐assisted extraction at 800 W.

Emulsifying capacity ranged from 600.68 to 621.31 mL oil g^−1^ OPNM, varying according to extraction condition, with the highest values observed in UAE400 and UAE800. These values were lower than those reported by Lise et al. (2021) for OPNM (2276.00 mL oil g^−1^). Such discrepancies might be due to differences in harvest period, leaf age, climate conditions, and extraction methods (Du Toit et al. 2019). Nevertheless, the emulsifying capacity of OPNM found here was similar to that of sodium caseinate (664 mL oil g^−1^), as reported by Ba et al. (2016). Sodium caseinate is widely used in food formulations, owing to its emulsifying, stabilizing, and texturizing capacity, which confirms the potential of OPNM for such purposes.

Emulsion stability ranged from 80.1% to 86.9% (w w^−1^), being higher in UAE400 and UAE800 samples. These values are similar to those reported by Lise et al. (2021) for OPNM (90%) and by Gemede et al. (2018) for Ethiopian okra mucilage (74.45%, w w^−1^).

UAE improved emulsifying capacity and emulsion stability. The technique alters protein structure, resulting in smaller particle sizes (lower molecular weight peptides) and greater exposure of hydrophobic groups, thereby improving emulsifying properties (Sun et al. 2020).

#### 3.6.3 Rheological Properties

The rheological properties of OPNM samples are shown in Table 5. UAE400 exhibited the highest apparent viscosity ($\eta_{\mathrm{ap}}=146.31\;\mathrm{mPa}\;s$), recorded at a shear rate of 300 s^−1^. The apparent viscosity was lower than that reported for linseed mucilage (204.8 mPa s) but higher than that of a mixture of the commercial thickeners maltodextrin and xanthan (88.2 mPa s) (Vilela et al. 2019).

The consistency index was highest in the CE25 sample (*K* = 7.08 Pa s*^n^*), followed by UAE400 (*K* = 4.89 Pa s*^n^*). Additionally, these OPNM samples exhibited the lowest fluidity indices, ranging from 0.25 (CE25) to 0.39 (UAE400), indicating a highly cohesive structure resistant to deformation. All OPNM samples were characterized as non‐Newtonian fluids and pseudoplastics, given that all had *n *< 1 (Pérez et al. 2021). This pseudoplastic behavior indicates that, as the shear rate increases, the initially randomly arranged polymer chains tend to align in the direction of flow, thereby reducing intermolecular interactions (Pérez et al. 2021).

Among OPNM samples obtained by UAE, the use of an intermediate power level (400 W) was particularly effective in maintaining high viscosity and structure (higher *k* and lower *n*). This behavior may be due to the fact that low power may not provide sufficient energy to disrupt cell structures and release polysaccharides effectively, and excessively high power may cause the degradation of polymer chains in mucilage (Wang et al. 2022).

#### 3.6.4 FTIR

**FIGURE 1:**
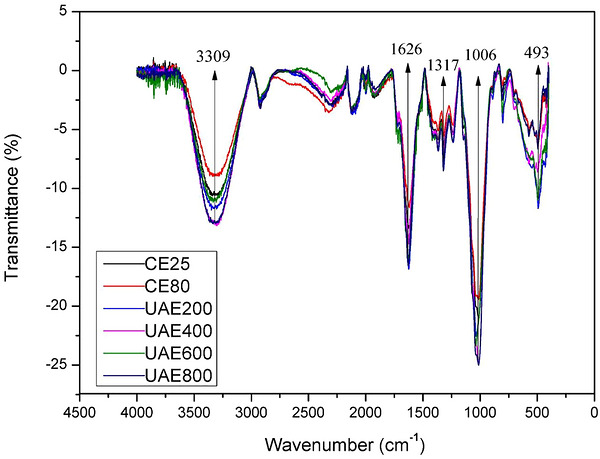
Fourier‐transform infrared spectra of ora‐pro‐nóbis mucilage obtained using different extraction methods. CE25, conventional extraction at 25°C; CE80, conventional extraction at 80°C; UAE200, ultrasound‐assisted extraction at 200 W; UAE400, ultrasound‐assisted extraction at 400 W; UAE600, ultrasound‐assisted extraction at 600 W; UAE800, ultrasound‐assisted extraction at 800 W.

Figure 1 shows the FTIR spectra of OPNM samples, which were similar between the applied treatments.

Table S1 details the vibrational mode assignments of OPNM samples. The wide bands occurring at 3500–3000 cm^−1^ in all samples indicate the presence of OH groups, related to carboxylic acids and polysaccharides (Rodriguez‐Navarro et al. 2017). Higher intensity peaks at 1626 cm^−1^ are attributed to stretching vibrations of the carboxylic group C═O, related to esterified galacturonic acids from pectin. The bands at 1400–1000 cm^−1^ indicate stretching vibrations of C–O (∼1317 cm^−1^) (Guo et al. 2013), and those at 1100–1000 cm^−1^ indicate the stretching vibrations of asymmetric ethers (∼1006 cm^−1^) (Silverstein et al. 2014). The peak at 493 cm^−1^ was associated with skeletal and network vibrations of polysaccharide structures (200 and 600 cm^−1^), suggestive of interactions within the polysaccharide matrix. These bands are characteristic of structured polysaccharides, such as cellulose and pectins (Nakamoto 2009).

Peak intensities varied little between samples, with the CE80 sample exhibiting lower peaks at 3309, 1317, and 493 cm^−1^. These regions are associated with O–H stretching, C–O vibrations, and C–O–C bond deformations, respectively, common features in polysaccharide and protein structures. The decrease in intensity may indicate structural modifications resulting from exposure to higher temperatures for a prolonged period, such as partial degradation of chains or rupture of bonds (Chen et al. 2025). Overall, the spectral patterns confirm the presence of polysaccharides and proteins, in particular, bands at 3309, 1626, and 1317 cm^−1^, which are the major components responsible for the emulsifying and gelling characteristics of OPNM (Yang et al. 2023). Additionally, similarities were found between the spectral bands of the OPNM samples obtained here and those reported by Nascimento et al. (2023) for lyophilized OPNM, with small differences in peak intensity.

#### 3.6.5 Scanning Electron Microscopy

**FIGURE 2:**
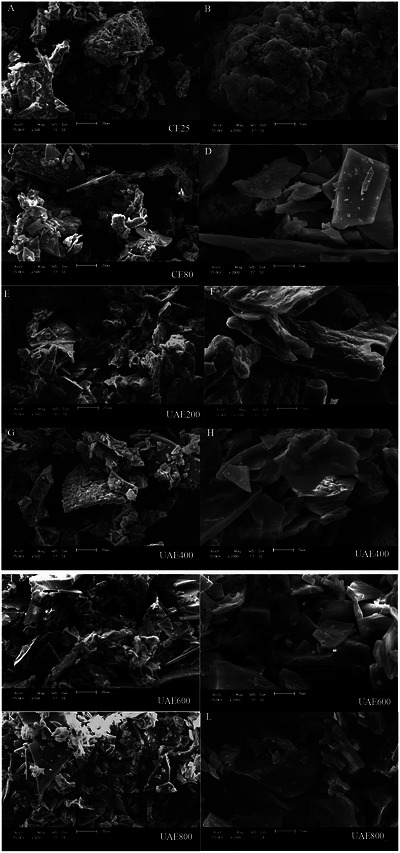
Scanning electron micrograph of ora‐pro‐nóbis mucilage obtained using different extraction methods. (A and B) CE25, conventional extraction at 25°C; (C and D) CE80, conventional extraction at 80°C; (E and F) UAE200, ultrasound‐assisted extraction at 200 W; (G and H) UAE400, ultrasound‐assisted extraction at 400 W; (I and J) UAE600, ultrasound‐assisted extraction at 600 W; and (K and L) UAE800, ultrasound‐assisted extraction at 800 W.

Figure 2 shows the micrographs of OPNM obtained by different extraction methods that were magnified by 500× and 2000×. The visible cracks represent deformities of the plate on which the samples were deposited for analysis. Overall, the samples had similar characteristics, with irregular and rough surfaces, suggesting high porosity. This particle morphology explains the high rates of water and oil absorption.

Particle size varied, and most particles exhibited a triangular shape, similar to that reported by Silva et al. (2024) for powdered OPN leaves. Nabil et al. (2019) argued that dehydration and mechanical crushing may contribute to particle non‐uniformity.

Some particles had a spherical shape, noticeable in all samples, albeit more expressively in CE25 (Figure 2A,B). According to Akbarbaglu et al. (2021), spherical particles with rough surfaces stem from the formation of films on the surface by substances such as polysaccharides during water evaporation. This morphology facilitates powder reconstitution in aqueous solutions, which improves the functionality of the final product, as confirmed by technological characterization (WAI, OAI, and emulsifying capacity).

#### 3.6.6 Amino Acid Profile

The OPNM sample obtained by UAE400 exhibited good results in most analyses, particularly in terms of technological properties and viscosity, which are essential for application in the food industry. Thus, the amino acid composition of this sample was determined (Table 6). This OPNM was found to contain all essential amino acids. Similarly, Lise et al. (2021) found a complete profile of essential amino acids in OPNM.

**TABLE 6:** Amino acid composition of ora‐pro‐nóbis mucilage obtained by ultrasound‐assisted extraction at 400 W.

Essential amino acids	Content (mg 100 g^−1^ mucilage)	Nonessential amino acids	Content (mg 100 g^−1^ mucilage)
Isoleucine	104.550 ± 1.040	Aspartic acid	327.230 ± 2.050
Valine	51.271 ± 1.901	Glutamic acid	72.671 ± 0.232
Methionine	51.220 ± 0.092	Alanine	60.550 ± 0.682
Histidine	992.330 ± 5.231	Tyrosine	83.262 ± 1.147
Leucine	313.483 ± 1.151	Serine	36.390 ± 0.207
Phenylalanine	142.011 ± 1.801	Glycine	86.675 ± 0.197
Lysine	265.651 ± 6.810	Arginine	210.310 ± 0.832
Tryptophan	182.561 ± 1.604	Asparagine	164.721 ± 0.450
Threonine	319.830 ± 0.434	Glutamine	202.722 ± 1.062

Among essential amino acids, leucine, threonine, and histidine were the major components. The latter had the highest concentration (992.33 mg 100 g^−1^). Histidine plays a crucial role in gastric acid secretion and regulation and exerts structural and catalytic functions in various enzymes. Additionally, it has anti‐inflammatory and antioxidant properties (Kessler and Raja 2023).

All nonessential amino acids (Table 6) were detected in OPNM (UAE400 sample), especially glutamine, arginine, and aspartic acid. Aspartic acid, found in a higher concentration, is fundamental for the body, as it participates in the citric acid cycle, urea cycle, and gluconeogenesis (Kumar et al. 2017); contributes to protein synthesis; and is involved in the biosynthesis of other amino acids, nucleotides, and hormones (Han et al. 2021).

## 4 Conclusion

The extraction methods evaluated in this study did not affect the mucilage yield of OPN leaves. Nevertheless, UAE improved the functional properties of the extracted material. OPNM extracted at 400 W showed superior water absorption, emulsifying properties, and rheological behavior, relevant characteristics for thickening and stabilization purposes in food systems. By contrast, high ultrasonic power (800 W) favored the recovery of phenolic compounds and antioxidant activity, indicating that extraction conditions may be selected according to the targeted application. OPNR retained considerable amounts of protein. Furthermore, the high oil absorption capacity of OPNR indicates its potential for oil retention, suggesting its applicability in improving texture, juiciness, and product stability, as well as serving as a partial fat replacer in food formulations. These findings demonstrate that OPN can be fractionated into ingredients with complementary functionalities, contributing to adding value to this underexplored plant material.

## Author Contributions

**Larissa Millena Girotto**: resources, methodology, formal analysis, writing – review and editing, writing – original draft, data curation, investigation. **Dayane Lilian Gallani Silva**: formal analysis, writing – review and editing. **Anderson Reginaldo Sampaio**: methodology, formal analysis, writing – review and editing. **Barbara Daniele Almeida Porciuncula**: methodology, writing – review and editing, supervision, formal analysis, conceptualization. **Beatriz Cervejeira Bolanho Barros**: funding acquisition, writing – original draft, writing – review and editing, visualization, validation, supervision, project administration, conceptualization.

## Conflicts of Interest

The authors declare no conflicts of interest.

## Supporting information




**Figure S1**: Mucilage extraction and residue obtention from ora‐pro‐nóbis leaves.
**Table S1**: Fourier‐transform infrared spectroscopy band assignments for ora‐pro‐nóbis mucilage samples.

## Data Availability

The data presented in the figures of this study are available upon request from the corresponding author.
