# Supplementary material for: Ultrasound‐Assisted Extraction of Mucilage: An Approach for the Full Utilization of Ora‐Pro‐Nóbis Leaves
Source: J Food Sci. 2026 Aug 17;91(8):e71373. doi: 10.1111/1750-3841.71373 (PMC13481829; doi:10.1111/1750-3841.71373)
Supplement: Supplementary file 1 [file JFDS-91-0-s001.docx]

**FIGURE S1.** Mucilage extraction and residue obtention from ora-pro-nóbis leaves.

**TABLE S1.** Fourier-transform infrared spectroscopy band assignments for ora-pro-nóbis mucilage samples.

| **Wavenumber (cm^−1^)** | **Functional group** | **Vibrational mode** | **Proposed assignment** | **References** |
| --- | --- | --- | --- | --- |
| ~3309 | O–H | Stretching (*ν* O–H) | Hydroxyl groups associated with polysaccharides, pectins, carboxylic acids, and bound water | Rodriguez-Navarro et al. (2017); Yang et al. (2023) |
| ~1626 | C=O/COO⁻ | Stretching (*ν* C=O) | Carbonyl groups from esterified galacturonic acid residues of pectin; possible contribution of carboxylate groups | Guo et al. (2013); Yang et al. (2023) |
| ~1317 | C–O | Stretching (*ν* C–O) | Alcohol and polysaccharide backbone vibrations, characteristic of pectin and cellulose structures | Guo et al. (2013) |
| ~1006 | C–O–C | Asymmetric stretching (*ν*_as_ C–O–C) | Glycosidic linkages and ether groups present in polysaccharides | Silverstein et al. (2014) |
| ~493 | C–O–C/skeletal vibrations | Deformation and network vibrations | Tentatively assigned to skeletal and network vibrations of polysaccharide structures | Nakamoto (2009) |
